# Association between serum uric acid and non-alcoholic fatty liver disease (NAFLD): an observational cross-sectional study in an Egyptian outpatient cohort

**DOI:** 10.1186/s12876-026-04655-2

**Published:** 2026-03-19

**Authors:** Haitham A. Mahmoud, W. Mohamed Abd Elghany, Mohammed Abdelhakeem, Hasnaa M. Ahmed, Nady Semeda, Safaa M Abdelhalim, Alaa M Mostafa, Shaimaa H Zaki, Manar M Sayed, Omar Abdelazim

**Affiliations:** 1 Department of Tropical Medicine, Minia University, Minia, Egypt; 2 Department of Clinical Pathology, Minia University, Minia, Egypt; 3 Department of Medical Microbiology and Immunology, Minia University, Minia, Egypt; 4 Department of Radiodiagnosis, Minia University, Minia, Egypt

**Keywords:** Non-alcoholic fatty liver disease, Serum uric acid level, Hyperuricemia, Hepatic steatosis

## Abstract

**Background:**

Hepatic steatosis, characterized by excessive triglyceride accumulation within hepatocytes, is the central feature of non-alcoholic fatty liver disease (NAFLD). This spectrum encompasses simple steatosis, non-alcoholic steatohepatitis (NASH), progressive fibrosis, and cirrhosis. NAFLD pathogenesis involves an intricate interplay between nutritional factors, metabolic dysregulation, and genetic predisposition. Evidence suggests hyperuricemia is an independent risk factor for NAFLD, with elevated serum uric acid (SUA) levels associated with increased steatosis severity and fibrosis advancement. This study investigated the association between SUA levels and liver involvement—specifically ultrasound-assessed steatosis grading and elastography-measured liver stiffness—in patients with NAFLD.

**Methods:**

This cross-sectional study enrolled 70 patients aged ≥18 years with ultrasound-diagnosed NAFLD (Group I) and 70 age-, sex-, and residence-matched healthy controls (Group II). Participants were recruited from Minia University Liver Hospital between March 2022 and February 2023. Exclusion criteria included excessive alcohol consumption, viral or autoimmune hepatitis, established cirrhosis, and medications affecting uric acid metabolism. The primary outcome was presence of NAFLD. Evaluations comprised detailed medical history, anthropometric measurements, laboratory testing (including SUA), abdominal ultrasonography for steatosis grading, and two-dimensional shear wave elastography for liver stiffness assessment.

**Results:**

Hyperuricemia prevalence reached 55.7% (*n*=39) among NAFLD patients compared to 0% in controls (*p*=0.001). Mean SUA concentrations were significantly elevated in the NAFLD group versus controls (5.98 ± 1.9 mg/dL vs. 4.34 ± 0.7 mg/dL, *p*=0.001). A significant association emerged between hyperuricemia and hepatic steatosis grades (*p*=0.032). Multivariate logistic regression identified SUA as independently associated with NAFLD after adjustment for confounders (Adjusted OR 1.45; 95% CI: 1.12–1.88, *p*=0.005 per 1 mg/dL increment), alongside BMI, waist circumference, triglycerides, and creatinine. Receiver operating characteristic (ROC) analysis demonstrated an optimal 5.3 mg/dL SUA cut-off for NAFLD discrimination (AUC 0.74, 64% sensitivity, 94% specificity, *p*=0.001).

**Conclusion:**

Elevated SUA levels were consistently associated with NAFLD presence, steatosis severity, and increased liver stiffness. The 5.3 mg/dL threshold warrants further validation. Notable limitations include single-center recruitment potentially limiting generalizability, a cross-sectional design precluding temporal relationship and causality determination, and the possibility of residual confounding from unmeasured variables.

**Supplementary Information:**

The online version contains supplementary material available at 10.1186/s12876-026-04655-2.

## Background

Hepatic steatosis involves the accumulation of fat, predominantly triglycerides, within liver tissue. In some countries, ultrasound evaluations indicate that non-alcoholic fatty liver disease (NAFLD) affects more than 25% of the general population [1].

Non-alcoholic fatty liver disease (NAFLD) presents with a range of conditions including simple steatosis, nonalcoholic steatohepatitis (NASH), fibrosis, and cirrhosis, which may advance hepatocellular carcinoma (HCC) and liver failure [2, 3]. The development of NAFLD is influenced by multiple factors such as nutrition, metabolism, and genetics [4]. Additionally, oxidative stress, insulin resistance, and systemic inflammation are important contributors to the initiation and progression of liver diseases including NAFLD and NASH [5, 6].

Obesity is closely associated with an increased risk of developing non-alcoholic fatty liver disease (NAFLD). Longitudinal studies indicate that individuals with obesity-related NAFLD frequently experience a decline in skeletal muscle mass over extended periods, a phenomenon termed sarcopenic obesity [7]. Alterations in fatty acid metabolism, commonly observed in aging and obesity, promote lipid accumulation within muscle tissue [8]. This process, known as myosteatosis, negatively impacts muscle strength by disrupting muscle fiber architecture, ultimately contributing to decreased mobility in older adults [9].

Multiple observational studies have demonstrated that hyperuricemia - characterized by serum uric acid (SUA) levels exceeding 7.0 mg/dL in men and 5.7 mg/dL in women - is linked to a heightened risk of non-alcoholic fatty liver disease (NAFLD) in Eastern Asian populations, independent of metabolic syndrome (MetS) factors [10, 11].

Uric acid is produced as a result of purine metabolism, with purines largely derived from increased intake of purine-rich foods such as meats and seafood, in addition to fructose and alcohol consumption [12]. Upon entering hepatocytes, fructose undergoes rapid phosphorylation that leads to a reduction in intracellular phosphate levels. This reduction activates adenosine monophosphate (AMP) deaminase, which causes substrate-dependent phosphate depletion and consequent adenosine triphosphate (ATP) depletion, thereby promoting increased uric acid synthesis [12, 13]. Some studies also indicate that depletion of hepatic ATP can inhibit protein synthesis and trigger inflammatory and pro-oxidative changes [14, 15].

Uric acid (UA), recognized for its potent antioxidant effects, has recently been implicated in sarcopenia [16]. Several studies have reported a significant inverse relationship between UA concentrations and sarcopenia incidence, likely due to UA’s antioxidant capabilities [17, 18, 19]. Oxidative stress is a key factor in age-related sarcopenia and involves excessive generation of reactive oxygen species [20, 21]. UA may support muscle health by neutralizing reactive oxygen species, thereby reducing oxidative damage [22].

If a causal relationship between hyperuricemia and NAFLD is established, managing hyperuricemia could help reduce the incidence or progression of NAFLD. Alternatively, if hyperuricemia is found to result from NAFLD, it may function as a clinical marker prompting healthcare professionals to investigate NAFLD and related liver conditions. In such cases, hyperuricemia could become a useful biomarker to identify individuals at elevated risk of NAFLD, independent of other known risk factors [23].

Additive interaction analysis demonstrated a significant synergistic effect between hyperuricemia and obesity in the progression of hepatic steatosis and fibrosis. In cases where both conditions co-occurred, 39% of severe hepatic steatosis and 60% of advanced fibrosis prevalence were attributable to the combined interaction [24]. The aim of this study was to examine the potential correlation between serum uric acid levels and the extent of liver damage in non-alcoholic fatty liver disease (NAFLD).

## Methods

### Study design

This research is an observational cross-sectional study. This study was designed and reported in accordance with the STROBE (Strengthening the Reporting of Observational Studies in Epidemiology) guidelines to ensure transparency and completeness in reporting observational research. Participants included patients with an ultrasound-based diagnosis of NAFLD (Group I) and healthy controls matched for age, sex, and residence (Group II). They were recruited from the outpatient clinics of the Internal Medicine and Tropical Medicine departments at Minia University Liver Hospital between March 2022 and February 2023. The study received approval from the Research and Ethics Committee of the Faculty of Medicine, Minia University, in compliance with local research governance guidelines. Informed consent was obtained from all individual participants. Data collection involved medical history, physical examinations (including anthropometric measurements), laboratory tests, and radiological assessments.

All subjects fulfilled the following criteria:

Inclusion criteria:

1. Age ≥ 18 years.
2. Both sexes were.

Exclusion criteria:

*Patients with one of the following conditions will be excluded*:

1. Excessive alcohol consumption (>20 gm/day in men and 10 gm/day in women).
2. Liver fat deposition caused by hepatitis B or C, autoimmune liver disease, liver cirrhosis, or other causes of liver disease.
3. Current or history (within the past 6 months) of administration of steatogenic medications or drugs that affect uric acid metabolism.

## Methods

Participants underwent clinical examinations in the morning after an overnight fast. The physical examination included anthropometric measurements of height, weight, waist and hip circumferences, as well as systolic and diastolic blood pressures. BMI was calculated by dividing weight in kilograms by height squared in meters (kg/m²). Obesity was defined as a BMI greater than 30 kg/m² [25]. Abdominal obesity was defined as a waist circumference greater than 102 cm in men and 88 cm in women [26].

Hip circumference was measured at the widest point of the buttocks. In men, hip circumference ranged from 94 to 105 cm, while in women, it ranged from 97 to 108 cm [27].

### Variable definitions and coding

- BMI: Calculated as weight (kg)/height² (m); analyzed both continuously (kg/m²) and categorically (normal: 18.5–24.9; overweight: 25-29.9; obesity: ≥30 kg/m² [WHO criteria]).
- Waist circumference: Measured at midpoint between lower rib and iliac crest; abdominal obesity defined as > 102 cm (men), > 88 cm (women) [NCEP ATP III criteria]; analyzed continuously (cm) and categorically.
- Hip circumference: Measured at widest gluteal point; reported continuously (cm) [reference ranges: men 94–105 cm, women 97–108 cm].
- Blood pressure: Systolic/diastolic measured by standardized sphygmomanometer after 5-min rest (mmHg); hypertension defined as ≥ 140/90 mmHg.
- Lipids: Total cholesterol, HDL-C, LDL-C, triglycerides measured enzymatically (all mg/dL); dyslipidemia coded per NCEP ATP III thresholds.
- Creatinine: Measured by Jaffe kinetic method (mg/dL); used to calculate eGFR (mL/min/1.73 m²) via CKD-EPI equation.
- 2D-SWE liver stiffness: Measured in kPa using validated cutoffs for fibrosis staging (F0: ≤7.1 kPa; F1: >7.1; F2: >7.8; F3: >8.0; F4: >11.5 kPa) [28].
- Steatosis grades: Ultrasound scale (0–3): Grade 0 (normal); Grade 1 (increased echogenicity vs. renal cortex); Grade 2 (obscured portal vein walls); Grade 3 (indistinguishable diaphragmatic outline).

### Laboratory tests

Venous blood samples were obtained following an overnight fasting period of eight hours. These samples were then divided into two tubes for further analysis:

#### EDTA-containing tube

Approximately 1 mL of blood was collected for the complete blood count (CBC) analysis.

#### Plain tube

Approximately 4 mL of blood were collected and allowed to clot before being centrifuged to obtain serum. The serum obtained was utilized for various investigations including fasting blood glucose, renal function tests, liver function tests, and complete lipid profile analysis.

Serum uric acid (SUA) levels were determined using the uricase-POD enzymatic colorimetric method.

### Hyperuricemia definition

Hyperuricemia was defined a priori using established sex-specific clinical cutoffs: > 7.0 mg/dL in men and > 6.0 mg/dL in women, based on standard laboratory reference ranges and clinical guidelines for asymptomatic hyperuricemia. These predefined thresholds were applied for categorical analysis of hyperuricemia prevalence. Separately, ROC curve analysis identified an exploratory 5.3 mg/dL cut-off optimized for NAFLD discrimination in this cohort (AUC 0.74), representing a data-driven threshold distinct from the prespecified hyperuricemia definition.

### Imaging methodology

#### I- abdominal real-time ultrasonography

The diagnosis of NAFLD requires assessing hepatic fat through imaging techniques or histology while excluding other causes of secondary fat accumulation, such as alcohol use or steatogenic drugs [29]. The gold standard for diagnosing NAFLD is liver biopsy, but it is invasive, not highly reproducible, and expensive [30]. Transient elastography, which measures liver stiffness and quantifies steatosis using controlled attenuation parameters with high accuracy, is an alternative but not widely accessible technique. It also requires technical expertise and is unreliable in patients with severe obesity and ascites [31, 32]. Consequently, ultrasound (US) examination is the most commonly performed procedure in clinical practice for diagnosing NAFLD [33]. Despite being easily reproducible and inexpensive, US has high interindividual variability [34].

### NAFLD diagnosis criteria and quality control

NAFLD cases (Group I) were defined as having Grade 1–3 hepatic steatosis on abdominal ultrasound according to standardized criteria: (1) increased hepatic echogenicity relative to the renal cortex (Grade 1); (2) enhanced hepatic echogenicity obscuring the echogenic walls of portal venous branches (Grade 2); or (3) marked echogenicity rendering the diaphragmatic and portal venous walls indistinguishable (Grade 3). All examinations were performed by a single radiologist with more than 10 years of experience in hepatobiliary imaging using the same ultrasound equipment. Assessments were blinded to serum uric acid levels and other clinical data, so the radiologist was unaware of participants’ exposure (SUA) and group allocation (NAFLD vs. controls). Inter-observer variability was not formally assessed because a single experienced operator performed all scans following a standardized protocol to minimize subjectivity.

### Histological and ultrasonograghic grading of hepatic steatosis [35]

Histological grading:

- Grade 0: Lipid droplets are present in fewer than 5% of hepatocytes.
- Grade 1: Lipid accumulation occurs in 6% to 33% of hepatocytes.
- Grade 2: Lipid droplets are observed in 34% to 66% of hepatocytes.
- Grade 3: More than 66% of hepatocytes exhibit lipid accumulation.

Ultrasonographic findings:

- Grade 0: Normal hepatic echo pattern is observed.
- Grade 1: Hepatic echogenicity is increased relative to the renal cortex.
- Grade 2: Enhanced hepatic echogenicity obscures the echogenic walls of portal venous branches.
- Grade 3: The increased hepatic echogenicity causes the diaphragmatic and portal venous walls to be indistinguishable on ultrasound.

#### II- two-dimensional shear wave elastography (2D-SWE)

The same ultrasound system and convex probe were used for 2D-SWE. The transducer was placed intercostally at the right lobe of the liver, targeting the right anterior hepatic segment at an ideal depth of 3 to 5 cm from the liver capsule (at least 1 cm deep). Major blood vessels were avoided. The left hepatic lobe was excluded to prevent interference from cardiac movement.

The size of the 2D-SWE sample box was approximately 3 × 3 cm. When possible, the patient was asked to hold their breath for 5–10 s. Elastography Propagation Maps were then acquired, displaying the derived liver stiffness in a color-coded format.

**Fig. 1:**
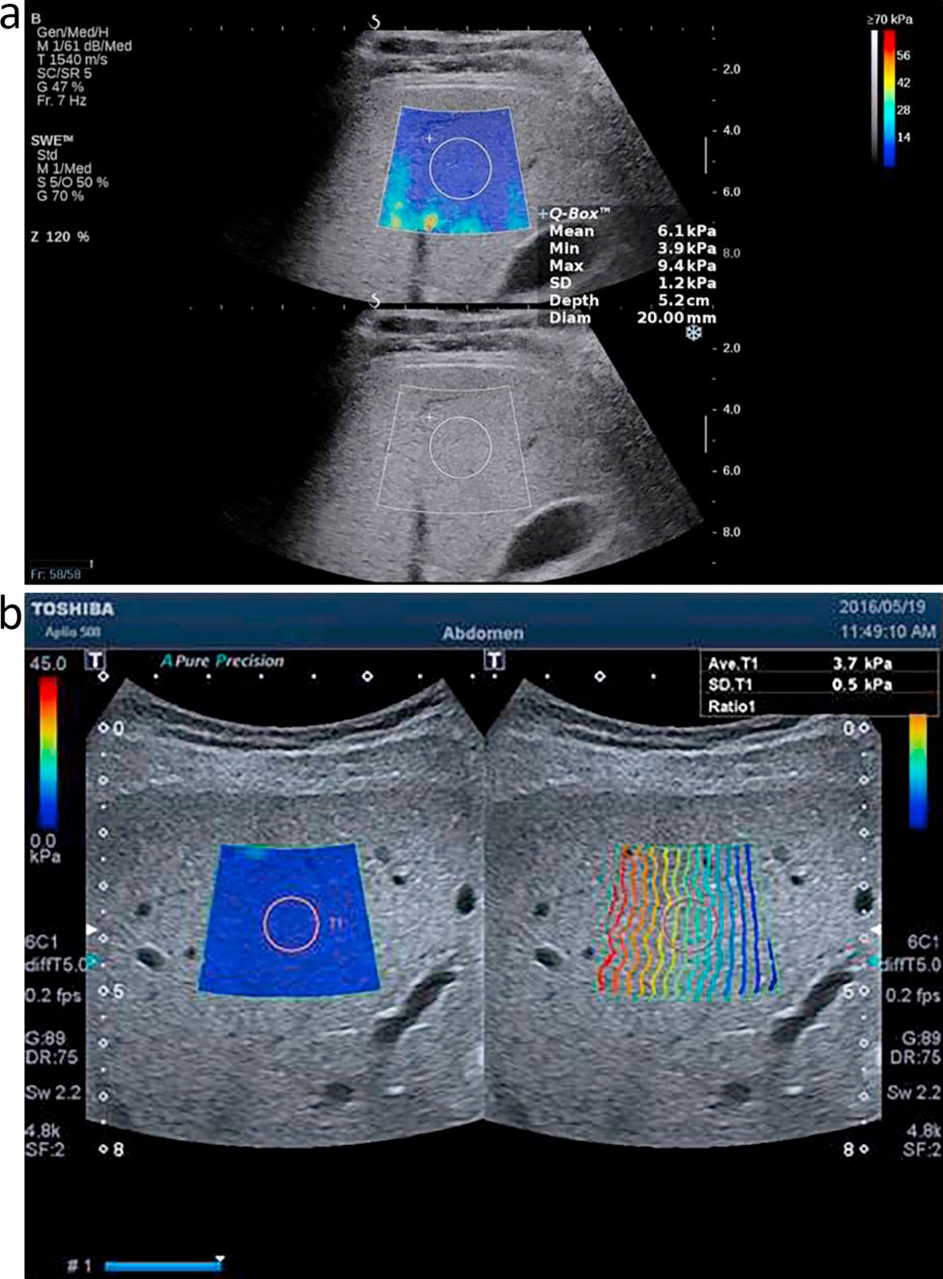
Normal liver parenchyma evaluated using 2D Shear wave elastography with different ultrasound systems. **a** Aixplorer (Supersonic Imagine). **b** Aplio 500 (Toshiba Medical Systems)

In addition to the qualitative visual assessment of these color charts, the quantitative region of interest (ROI)-based measurement of the SWE in meters per second (m/s) was obtained. The ROI was placed at the center of the target sample box. Measurements were performed at least five times, and the average of these measurements was used as the SWE value (mean ± standard deviation [SD]) (Table 1, Fig. 1). The radiologist performing 2D-SWE was blinded to serum uric acid levels, laboratory results, and case-control status.

**Table 1:** Cut-off values for different stages of liver fibrosis [28]

Fibrosis	2D-SWE Cut-off (kPa)
F1	> 7.1
F0	≤ 7.1
F2	> 7.8
F3	> 8
F4	> 11.5

### Use of 2D-SWE as a fibrosis surrogate

Two-dimensional shear wave elastography (2D-SWE) was used as a non-invasive surrogate for liver fibrosis assessment. Liver stiffness measurements were expressed in kilopascals (kPa) and categorized into fibrosis stages according to published cut-offs: F0 ≤ 7.1 kPa, F1 > 7.1–7.7 kPa, F2 > 7.8–7.9 kPa, F3 ≥ 8.0-11.5 kPa, F4 > 11.5 kPa [28]. These thresholds were derived from prior validation studies performed on the same 2D-SWE platform and probe model and have been applied to populations with similar etiologies and BMI distributions to our cohort. Fibrosis was analyzed as an ordinal variable (F0-F4) using one-way ANOVA and chi-square tests for group comparisons.

### Sample size calculation

The required sample size was calculated using G*Power software (version 3.1.9.7), based on a previous study by Oral et al. [36], which reported mean serum uric acid levels of 4.36 ± 1.36 mg/dL in NAFLD patients compared to 3.48 ± 1.30 mg/dL in healthy controls. Assuming a two-tailed t-test with 80% statistical power, a significance level of 0.05, and a medium effect size of 0.48, the minimum total sample size required was determined to be 140 participants (70 per group).

### Bias control measures

Measurement bias: All participants fasted ≥ 8 h overnight (confirmed by patient interview and scheduled morning appointments). Serum uric acid was measured using the same uricase-POD enzymatic colorimetric assay platform for all samples (intra-assay coefficient of variation [CV] < 2%, inter-assay CV < 3%). Anthropometric measurements followed standardized protocols by trained personnel using calibrated equipment.

Selection bias: Controls (Group II) were consecutively recruited from the same outpatient clinics using identical inclusion/exclusion criteria as NAFLD cases, matched for age (± 5 years), sex, and residence. Non-response rate was < 5% (3/73 eligible controls declined participation due to time constraints).

Blinding: Ultrasound and 2D-SWE assessments were performed by operators who were blinded to serum uric acid levels and to group allocation (NAFLD vs. control) to reduce measurement and diagnostic suspicion bias.

### Statistical analysis

- The data was examined with the use of SPSS, Version 25, which is a statistical package for the social sciences. Office 2010’s Excel was used to create the graphics.
- The frequency distribution was used to display qualitative data, whilst the mean and standard deviation were used to display quantitative data.
- To compare non-parametric quantitative data between the two groups, the Mann Whitney test was utilized. For parametric quantitative data, the independent sample t test was employed.
- When comparing parametric quantitative data across more than two groups, one way ANOVA was employed.
- When there were more than two groups involved in comparing qualitative data, the chi-square test was employed.
- To establish a connection between two numerical variables, the Pearson correlation was employed.
- A linear relationship between two variables can be defined by looking at their correlation coefficient, which is represented by the symbol r: Weak or no correlation is indicated by r grades of 0.00 to 0.24, fair correlation by 0.25 to 0.49, moderate correlation by 0.50 to 0.74, and strong correlation by > 0.75.
- The diagnostic test and other tests’ cut-off points were determined using ROC curve analysis.
- Every statistically significant test was considered to have passed if the probability was less than 0.05.

## Results

All subjects were divided into two groups:

- Group I: NAFLD group included 70 patients [16 (22.9%) males and 54 (77.1%) females].
- Group II: Control group included 70 healthy subjects matched for age, sex and residence [10 (14.3%) males and 60 (85.7%) females].

The demographic data and anthropometric measures of the studied groups are shown in Table 2. There was a statistically significant difference between NAFLD patients and healthy controls concerning BMI and waist and hip circumferences (*p* value: 0.001, 0.001, 0.001, respectively). However, there was no statistically significant difference between the two groups concerning age, sex, and residence (*p* value: 0.201, 0.192, and 0.839, respectively).

**Table 2:** Demographic data and anthropometric measures of the studied groups (*N* = 140)

Variable	Group I NAFLD (*N* = 70) *N* (%)	Group II Controls (*N* = 70) *N* (%)	*P* value
Age			0.201
Range (years)	19–65	21–60	
Mean ± SD	41.4 ± 11.2	39.2 ± 9.8	
Sex			0.192
Males	16 (22.9%)	10 (14.3%)	
Females	54 (77.1%)	60 (85.7%)	
Residence			0.839
Rural	15 (21.4%)	16 (22.9%)	
Urban	55 (78.6%)	54 (77.1%)	
BMI			0.001^*^
Range (kg/m^2^)	23.8–44.8	20.8–29.1	
Mean ± SD	30.8 ± 5	24.9 ± 2.3	
Waist circumference			0.001^*^
Range (cm)	72–144	68–105	
Mean ± SD	108 ± 11.8	89.6 ± 10.5	
Hip circumference			0.001^*^
Range (cm)	73–162	95–120	
Mean ± SD	112.9 ± 12.1	103.5 ± 6.1	

The asterisk (*) denotes statistical significance with a *p*-value < 0.05

The grades of hepatic steatosis and degrees of fibrosis for the studied groups are presented in Table 3. The majority of NAFLD patients exhibited grade 1 hepatic steatosis [38 (54.3%)] and F2 degree of fibrosis [40 (57.1%)]. Conversely, all (100%) controls had grade 0 hepatic steatosis and F0 degree of fibrosis. There was a statistically significant difference between the two groups concerning the grade of hepatic steatosis and the degree of fibrosis (*p* value: 0.001 and 0.001, respectively).

**Table 3:** Grades of hepatic steatosis and degrees of fibrosis of the studied groups (*N* = 140*)*

Variable	Group I NAFLD (*N* = 70) *N* (%)	Group II Controls (*N* = 70) *N* (%)	*P* value
Grade of steatosis by US			0.001^*^
Grade 0	0 (0%)	70 (100%)	
Grade 1	38 (54.3%)	0 (0%)	
Grade 2	21 (30%)	0 (0%)	
Grade 3	11 (15.7%)	0 (0%)	
Degree of fibrosis			0.001^*^
F0	12 (17.1%)	70 (100%)	
F1	10 (14.3%)	0 (0%)	
F2	40 (57.1%)	0 (0%)	
F3	8 (11.5%)	0 (0%)	

The asterisk (*) denotes statistical significance with a *p*-value < 0.05

Serum uric acid levels of the studied groups are presented in Table 4. The mean ± SD serum uric acid level was higher in NAFLD patients (5.98 ± 1.9) compared to controls (4.34 ± 0.7), showing a statistically significant difference (*p* value: 0.001). Among NAFLD patients, 39 (55.7%) were hyperuricemic, while 31 (44.3%) were normouricemic. All controls were normouricemic, with a statistically significant difference between the two groups (*p* value: 0.001).

**Table 4:** Serum uric acid level of the studied groups (N=140)

Serum uric acid level	Group I NAFLD (*N* = 70) *N* (%)	Group II Controls (*N* = 70) *N* (%)	*P* value
Range (mg/dL)	2.1–9	2.3–5.3	0.001^*^
Mean ± SD	5.98 ± 1.9	4.34 ± 0.7	
Hyperuricemia	39 (55.7%)	0 (0%)	0.001^*^
Normouricemia	31 (44.3%)	70 (100%)	

The asterisk (*) denotes statistical significance with a *p*-value < 0.05

The relation between the grades of hepatic steatosis, degrees of fibrosis, and hyperuricemia in patients with NAFLD is shown in Table 5. We found a statistically significant relation between hyperuricemia and the grades of hepatic steatosis by ultrasound (*p* value: 0.032).

**Table 5:** Relation between the grades of hepatic steatosis, the degrees of fibrosis and hyperuricemia in patients with NAFLD (*N* = 70)

Variable	Normouricemia (*N* = 31) *N* (%)	Hyperuricemia (*N* = 39) *N* (%)	*P* value
Degree of fibrosis			0.109
F0	4 (12.9%)	8 (20.5%)	
F1	5 (16.2%)	5 (12.9%)	
F2	20 (64.5%)	20 (51.3%)	
F3	2 (6.5%)	6 (15.4%)	
Grade of steatosis by US			0.032^*^
Grade 1	22 (71%)	16 (41%)	
Grade 2	7 (22.6%)	14 (35.9%)	
Grade 3	2 (6.5%)	9 (23.1%)	

The asterisk (*) denotes statistical significance with a *p*-value < 0.05

The correlation between the components of metabolic syndrome and serum uric acid level in patients with NAFLD is shown in Table 6. There was a significant correlation between waist circumference and serum uric acid level in NAFLD patients (*p* value: 0.022). However, neither systolic blood pressure (SBP), diastolic blood pressure (DBP), fasting blood glucose (FBG), serum triglycerides, nor high density lipoproteins (HDL) had a statistically significant correlation with SUA level in NAFLD patients (*p* values: 0.334, 0.281, 0.096, 0.517, and 0.287, respectively).

**Table 6:** Correlation between the components of metabolic syndrome and serum uric acid level in patients with NAFLD (*N* = 70)

Variable	Serum uric acid level in NAFLD patients (*N* = 70)
Waist circumference	
r	0.273
p	0.022^*^
SBP	
r	0.117
p	0.334
DBP	
r	0.131
p	0.281
FBG	
r	0.200
p	0.096
Triglycerides	
r	0.079
p	0.517
HDL	
r	0.129
p	0.287

The asterisk (*) denotes statistical significance with a *p*-value < 0.05

Logistic regression analysis for factors associated with NAFLD among all the studied groups is presented in Table 7. Univariate analysis revealed a significant association between NAFLD and the following factors: BMI, waist circumference, serum triglycerides, SBP, blood urea, serum creatinine, and serum uric acid. Additionally, multivariate logistic regression analysis indicated a significant association between NAFLD and the following factors: BMI, waist circumference, serum triglycerides, serum creatinine, and serum uric acid.

**Table 7:** Logistic regression analysis for factors associated with NAFLD among all the studied groups (*N* = 140)

Independent variables	Univariate analysis		Multivariate analysis	
	Crude OR (95% confidence interval [CI])	*P* value	Adjusted OR^*^ (95% CI)	*P* value
Age	1.021 (0.98–1.05)	0.200	0.980 (0.92–1.05)	0.574
Sex	0.563 (0.235–1.345)	0.196	0.199 (0.037–1.05)	0.057
BMI	1.73 (1.44–2.07)	0.001^*^	1.72 (1.7–1.2)	0.006^*^
Waist circumference	1.243 (1.14–1.345)	0.001^*^	1.14 (1.04–1.24)	0.004^*^
SBP	1.067 (1.03–1.9)	0.001^*^	1.03 (0.95–1.11)	0.424
FBG	1.01 (0.99–1.03)	0.154	1.03 (0.97–1.08)	0.329
Triglycerides	1.038 (1.02–1.05)	0.001^*^	1.04 (1.01–1.07)	0.011^*^
HDL	0.954 (0.86–1.01)	0.146	0.932 (0.81–1.07)	0.317
Urea	1.05 (1.01–1.08)	0.007^*^	0.943 (0.84–1.05)	0.316
Creatinine	20.6 (19.6–196)	0.001^*^	10.9 (8–43)	0.006^*^
Uric acid	2.235 (1.63–3.06)	0.001^*^	2.3 (1.06–5.014)	0.030^*^

The asterisk (*) denotes statistical significance with a *p*-value < 0.05

### Sensitivity and specificity of serum uric acid for diagnosis of NAFLD

Serum uric acid was statistically significant in diagnosing NAFLD at a cut-off value of 5.3 with AUC of 74%, sensitivity of 64% and specificity of 94% (p value: 0.001).

**Fig. 2:**
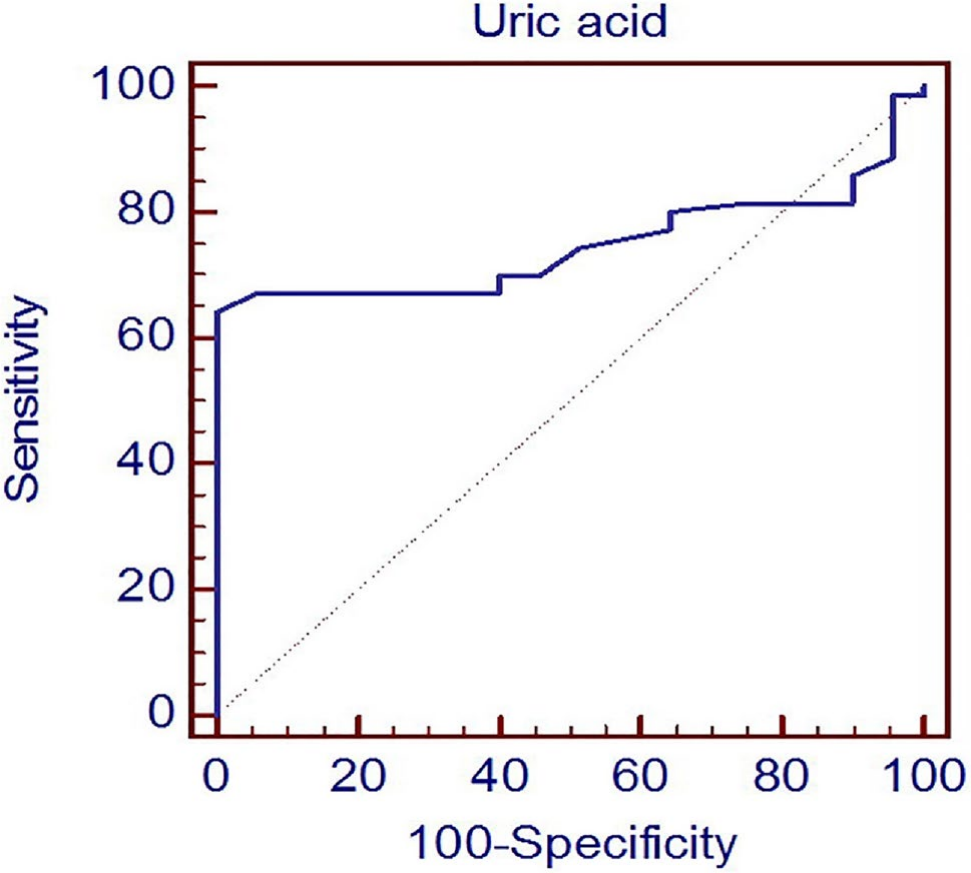
ROC curve analysis of serum uric acid level for diagnosis of NAFLD

ROC curve analysis of serum uric acid level for diagnosis of NAFLD was shown in Fig. 2.

The results are illustrated in the following tables and figures.

## Discussion

Non-alcoholic fatty liver disease (NAFLD) has emerged as a significant concern in public health due to its widespread occurrence [37]. Studies suggest that its frequency ranges from 20 to 30% in Western nations and affects 15–30% of the overall population in China [38]. NAFLD is frequently linked with conditions such as obesity, type 2 diabetes mellitus, dyslipidemia, and hypertension, and is also viewed as a liver-related component of metabolic syndrome (MetS) [39].

Much like NAFLD, serum uric acid (SUA), the byproduct of purine metabolism by the liver, has also been implicated in both metabolic syndrome (MetS) and cardiovascular disease [40]. Various pieces of evidence suggest that elevated serum uric acid (SUA) is often linked to the onset or progression of NAFLD [41]. A previous epidemiological investigation indicated that SUA serves as an independent risk factor for cardiovascular diseases, with insulin resistance being a significant contributing factor to NAFLD development. Sirota et al. [41] illustrated that heightened uric acid levels are independently correlated with ultrasound-diagnosed NAFLD, irrespective of insulin resistance.

In this study, we aimed to:

1. Determine the association between ultrasound-defined NAFLD and SUA.
2. Determine if hyperuricemia is associated with severity of liver damage.

The research conducted is an observational cross-sectional study. It involved patients diagnosed with NAFLD based on ultrasound findings (group 1), as well as a control group consisting of healthy individuals matched for age and sex (group 2). Group 1 participants were recruited from the outpatient clinics of the Internal Medicine and Tropical Medicine departments at Minia University Liver Hospital between March 2022 and February 2023.

The patient groups diagnosed with NAFLD, and the control cohorts underwent comprehensive history assessments and clinical examinations. Anthropometric parameters such as BMI, waist circumference, and hip circumference were measured for both patients and controls. Laboratory analyses, including complete blood counts, liver function tests, renal function tests, fasting blood sugar levels, lipid profiles, and serum uric acid levels, were conducted for all participants. Radiological assessments, including abdominal ultrasound examinations, were utilized for diagnosing and evaluating the extent of steatosis, while two-dimensional shear wave elastography (2D-SWE) was employed to assess the degree of hepatic tissue fibrosis.

In our investigation, we observed no notable distinction in demographic characteristics, including age, sex, and residence, between patients diagnosed with NAFLD and the control group (p values: 0.201, 0.192, and 0.839, respectively). Despite a higher proportion of females in both the NAFLD patient group and the control group compared to males (77.1% vs. 22.9% and 85.7% vs. 14.3%, respectively), this contrast did not reach statistical significance (*p* value: 0.192). However, Hwang et al. [42] noted that the mean age and the proportion of males were significantly greater in patients with NAFLD compared to controls (75.9% vs. 43.8%, *p* value: <0.001). Additionally, Rahman et al. [43] reported a higher prevalence of NAFLD in males compared to females.

Additionally, our analysis revealed that the average values of anthropometric measurements, including BMI, waist circumference, and hip circumference, were significantly elevated in patients diagnosed with NAFLD compared to the control group (p value: 0.001). This finding aligns with the research conducted by Oral et al. [36], who studied 225 patients with histologically confirmed NAFLD alongside 142 controls without NAFLD. They observed that the mean BMI among NAFLD patients was 27.25 ± 4.02, significantly higher than the mean BMI of 24.71 ± 3.34 among controls (*p* value: 0.001). Similarly, Kosekli et al. [44] demonstrated that the median hip circumference levels were significantly greater in patients with NAFLD compared to controls [10 (90–157) vs. 100 (75–126), *p* value: 0.001].

Concerning the ultrasonographic assessment of hepatic steatosis severity among patients diagnosed with NAFLD, our findings indicate that the majority fell into grade I [38 (54.3%)], followed by grade II [21 (30%)] and grade III [11 (15.7%)] hepatic steatosis. Among the NAFLD patients, most were classified as F2 [40 (57.1%)], followed by F0, F1, and F3 [12 (17.1%), 10 (14.3%), and 8 (11.5%), respectively] degree of fibrosis.

Likewise, the research conducted by Zenovia et al. [45] revealed that a large portion of NAFLD patients exhibited either no liver fibrosis [F0: 73 (40.3%)] or only mild fibrosis [F1: 42 (23.2%)]. Conversely, the proportion of patients with significant fibrosis [F2: 32 (17.7%)], advanced fibrosis [F3: 19 (10.5%)], and cirrhosis [F4: 15 (8.3%)] was relatively lower. Additionally, they noted a significant increase in liver stiffness measurement (LSM) values corresponding with the severity of steatosis (S0 to S3, *p* value: <0.001).

Hernaez et al. [46] stated that ultrasound provides a dependable and precise means of identifying moderate to severe fatty liver compared to histology. Conversely, two-dimensional shear wave elastography (2D-SWE) has emerged as a non-invasive technique for assessing liver tissue elasticity in clinical settings. A primary advantage of 2D-SWE, in contrast to transient elastography, is its integration with conventional grey-scale ultrasound probes, enabling practical use in various clinical scenarios. Additionally, it permits the operator to select a homogeneous portion of liver parenchyma, thus avoiding focal lesions [47].

Regarding serum uric acid levels, our study also found that the mean ± SD serum uric acid level was significantly higher in patients diagnosed with NAFLD compared to the control group (5.98 ± 1.9 vs. 4.34 ± 0.7, *p* value: 0.001). Among NAFLD patients, thirty-nine (55.6%) exhibited hyperuricemia. These findings are consistent with those reported by Oral et al. [36], who observed significantly higher serum uric acid levels in NAFLD patients compared to controls (4.36 ± 1.36 vs. 3.48 ± 1.30, *p* value: <0.001).

During our investigation, we identified a statistically significant relation between the grades of hepatic steatosis detected by ultrasonography and the presence of hyperuricemia (*p* value: 0.032). Furthermore, our study revealed a statistically significant correlation between serum uric acid levels and waist circumference (p value: 0.022).

These findings are consistent with the observations made by Lee et al. [10], who noted that serum uric acid levels increased in accordance with the severity of hepatic steatosis: 327.4, 349.5, 362.5, and 375.9 mmol/L in men, and 235.3, 257.8, 274.0, and 314.9 mmol/L in women with normal, mild, moderate, and severe fatty liver, respectively. Similarly, Zheng et al. [48] reported that individuals with fatty liver exhibited significantly elevated serum uric acid levels compared to those without fatty liver. Moreover, patients with moderate and severe fatty liver demonstrated notably higher serum uric acid levels than those with mild fatty liver, irrespective of gender.

The visceral fat component is metabolically active and plays a role in regulating various adipocytokines, including leptin and adiponectin, which are linked to insulin resistance. Insulin resistance or hyperinsulinemia can increase sodium and uric acid reabsorption in the renal tubules, leading to reduced urinary excretion of uric acid and potentially resulting in hyperuricemia [49, 50]. Elevated uric acid levels can upregulate the messenger ribonucleic acid (mRNA) expression of monocyte chemotactic protein-1 (MCP-1) while downregulating the mRNA expression of adiponectin. There exists a strong positive correlation between serum leptin and uric acid levels in both diabetic and healthy individuals [51]. Moreover, in patients with non-alcoholic fatty liver disease (NAFLD), hyperuricemia has been linked to more severe liver damage [52, 53].

We observed a significant correlation between NAFLD and several factors, including BMI, waist circumference, and serum levels of triglycerides, creatinine, and uric acid (*p* values: 0.006, 0.004, 0.011, 0.006, and 0.030, respectively). These findings align with the research conducted by Oral et al. [36], who performed a regression analysis with NAFLD/non-NAFLD as the binary outcome to assess the independence of associations between NAFLD and anthropometric and biochemical parameters. Their analysis revealed that the following factors were independently associated with NAFLD: BMI, homeostatic model assessment for insulin resistance (HOMA-IR), gamma-glutamyl transferase (GGT), and uric acid levels.

The elevation of serum uric acid levels is strongly linked to the accumulation of visceral fat and the initiation of hepatic histological alterations independently. Thus, as a significant risk factor, elevated uric acid levels may emerge as predictive markers for the occurrence and severity of NAFLD. This suggests that uric acid levels could be a promising therapeutic target for NAFLD, particularly in individuals with hyperuricemia [51].

Elevated uric acid levels can contribute to endothelial dysfunction, insulin resistance, oxidative stress, and systemic inflammation [54, 55], all of which are implicated in the pathogenesis of NAFLD [56].

In our study, we identified a cut-off value for uric acid of 5.3 mg/dl with an AUC of 74%, sensitivity of 64%, and specificity of 94% for the detection of NAFLD. This suggests that uric acid levels can serve as a predictive factor for the presence of NAFLD. In comparison, Oral et al. [36] found a cut-off value for serum uric acid (SUA) of 4.75 mg/dL with an AUC of 0.682, sensitivity of 45.8%, and specificity of 80.3% for predicting NAFLD. Wei et al. [57] reported the best cut-off value for SUA to predict NAFLD incidence as ≥ 288.5 µmol/L, with an AUC of 0.637, sensitivity of 75.5%, and specificity of 46.5% in total. They also provided gender-specific cut-off values: ≥319.5 µmol/L for males [AUC (95% CI): 0.590, sensitivity: 65.8%, specificity: 48.4%] and ≥ 287.5 µmol/L for females [AUC (95% CI): 0.662, sensitivity: 51.0%, specificity: 75.6%]. Furthermore, Zheng et al. [48] found an AUC of 0.70 for detecting mild fatty liver based on SUA (optimal cut-off value: 341.05 µmol/L, sensitivity: 70.7%, specificity: 59.9% and Youden index: 0.31), and an AUC of 0.78 for detecting moderate to severe fatty liver (optimal cut-off value: 370.15 µmol/L, sensitivity: 73.7%, specificity: 71.2% and Youden index 0.45). These studies collectively highlight the potential utility of uric acid as a diagnostic marker for NAFLD, albeit with variations in cut-off values and diagnostic accuracies across different populations and study settings.

### Limitations of the study

Further studies are needed to evaluate the relationship between sarcopenia and NAFLD and the effect of serum uric acid levels on muscle mass and peak muscle strength. Additionally, other factors such as the diet and physical activity of the enrolled patients should be analyzed in subsequent studies. It is important to note that our sample size was relatively small.

## Conclusion

There was a significant relation observed between serum uric acid (SUA) levels and NAFLD, which remained notable even after accounting for multiple metabolic risk factors. Furthermore, we noted a significantly higher prevalence of NAFLD among individuals with hyperuricemia compared to those without.

If hyperuricemia is established to be a causative factor in the development of NAFLD, then interventions aimed at preventing or treating hyperuricemia could potentially lower the risk of NAFLD formation or progression.

Uric acid emerges as a straightforward, non-invasive, cost-effective marker that holds utility in predicting the degree of hepatic steatosis and the severity of liver fibrosis in NAFLD patients. Moreover, we recommend exercising caution regarding NAFLD progression when serum uric acid levels exceed 5.6 mg/dL.

## Supplementary Information

Supplementary Material 1.

## Data Availability

No datasets were generated or analysed during the current study.

## Abbreviations

2D-SWE: Two-dimensional shear wave elastography
AMP: Adenosine monophosphate
ATP: Adenosine triphosphate
AUC: Area under the curve
BMI: Body mass index
CI: Confidence interval
DBP: Diastolic blood pressure
FBG: Fasting blood glucose
GGT: Gamma-glutamyl transferase
HCC: Hepatocellular carcinoma
HDL: High density lipoproteins
HOMA-IR: Homeostatic model assessment for insulin resistance
NAFLD: Non-alcoholic fatty liver disease
LSM: Liver stiffness measurement
NASH: Nonalcoholic steatohepatitis
MCP-1: Monocyte chemotactic protein-1
MetS: Metabolic syndrome
mRNA: Messenger ribonucleic acid
ROI: Region of interest
SBP: Systolic blood pressure
SD: Standard deviation. SUA: Serum uric acid
US: Ultrasoud
